# Air pollution & the brain: Subchronic diesel exhaust exposure causes neuroinflammation and elevates early markers of neurodegenerative disease

**DOI:** 10.1186/1742-2094-8-105

**Published:** 2011-08-24

**Authors:** Shannon Levesque, Michael J Surace, Jacob McDonald, Michelle L Block

**Affiliations:** 1 Department of Anatomy and Neurobiology, Virginia Commonwealth University Medical Campus, Richmond, VA 23298, USA; 2 Lovelace Respiratory Research Institute, Albuquerque, NM, 87108, USA

**Keywords:** Air pollution, diesel exhaust, midbrain, Tau hyperphosphorylation, α synuclein, TNFα, Aβ42

## Abstract

**Background:**

Increasing evidence links diverse forms of air pollution to neuroinflammation and neuropathology in both human and animal models, but the effects of long-term exposures are poorly understood.

**Objective:**

We explored the central nervous system consequences of subchronic exposure to diesel exhaust (DE) and addressed the minimum levels necessary to elicit neuroinflammation and markers of early neuropathology.

**Methods:**

Male Fischer 344 rats were exposed to DE (992, 311, 100, 35 and 0 μg PM/m^3^) by inhalation over 6 months.

**Results:**

DE exposure resulted in elevated levels of TNFα at high concentrations in all regions tested, with the exception of the cerebellum. The midbrain region was the most sensitive, where exposures as low as 100 μg PM/m^3 ^significantly increased brain TNFα levels. However, this sensitivity to DE was not conferred to all markers of neuroinflammation, as the midbrain showed no increase in IL-6 expression at any concentration tested, an increase in IL-1β at only high concentrations, and a decrease in MIP-1α expression, supporting that compensatory mechanisms may occur with subchronic exposure. Aβ42 levels were the highest in the frontal lobe of mice exposed to 992 μg PM/m^3 ^and tau [pS199] levels were elevated at the higher DE concentrations (992 and 311 μg PM/m^3^) in both the temporal lobe and frontal lobe, indicating that proteins linked to preclinical Alzheimer's disease were affected. α Synuclein levels were elevated in the midbrain in response to the 992 μg PM/m^3 ^exposure, supporting that air pollution may be associated with early Parkinson's disease-like pathology.

**Conclusions:**

Together, the data support that the midbrain may be more sensitive to the neuroinflammatory effects of subchronic air pollution exposure. However, the DE-induced elevation of proteins associated with neurodegenerative diseases was limited to only the higher exposures, suggesting that air pollution-induced neuroinflammation may precede preclinical markers of neurodegenerative disease in the midbrain.

## Background

Accumulating evidence points to neuroinflammation as an active participant in the progression of neurodegenerative diseases, such as Parkinson's disease (PD) and Alzheimer's disease (AD) [1-3]. In fact, current theory holds that pro-inflammatory events in the brain very likely occur across an individual's lifespan to culminate in neuropathology [3,4]. While environmental factors are largely implicated in the etiology of neurodegenerative disease [5,6], at present the various sources responsible for the chronic neuroinflammation leading to central nervous system (CNS) pathology are poorly understood.

Air pollution is a mixture comprised of several components, including particulate matter (PM, the particle components of air pollution), gases, and metals, such as vanadium, nickel, and manganese [7,8]. This toxin is readily available in the environment in many forms from multiple sources [8,9] and exposure occurs across and individual's entire lifetime. In fact, in the US alone, millions of people are exposed to levels of air pollution above established safety standards [8,10]. This is of significant concern, as diverse forms of air pollution have been widely implicated in inflammation and oxidative stress in humans [11].

While the majority of studies focus on the effects of air pollution in cardiovascular and pulmonary disease [12], accumulating evidence now points to a new role for air pollution in CNS disease [10]. For example, human studies have shown that living in conditions with elevated air pollution is associated with decreased cognitive function [13], AD-PD like neuropathology [14], and increased stroke incidence [15]. Even the individual air pollution components such as manganese have been linked to CNS pathology, as elevated levels of manganese in the air are linked to enhanced PD risk [16]. Consistent with human reports, recent animal studies reveal that exposure to diverse forms of air pollution by inhalation, such as urban PM [17,18], ozone [19], DE, and manganese [20,21] results in a common pro-inflammatory response and oxidative stress in the brain. However, given the significant expense of inhalation exposure studies, the majority of this experimental work is based on short term (one month - 10 weeks) studies, with only high exposure levels tested. While these studies are critical for understanding how air pollution affects the brain, human exposures to air pollution typically occur at lower concentrations. More specifically, PM levels in polluted US cities peak around 50 μg PM/m^3 ^[8], near-road PM concentrations are measured around approximately 100 μg PM/m^3^, and occupational exposure to PM occurs around 1000 - 2000 μg PM/m^3 ^[22,23], where human exposure continues for years.

Diesel exhaust (DE) is a form of air pollution that has received significant attention regarding its potential effect on human health in both ambient and occupational exposure conditions [24], and several studies have documented the CNS effects of DE. For example, acute, high level DE exposure affects electroencephalogram parameters in adult human subjects [25]. Animal research has shown that the prenatal period is a critical period of vulnerability, where maternal DE exposure affects dopamine neurochemistry and causes motor deficits in offspring [26,27]. Short term studies in young adult animals also demonstrate that DE elevates pro-inflammatory factors in the brain, using a month-long inhalation models [18,28], intratracheal administration directly into the lung [18], and a 2 hr-long exposure by nose-only inhalation [29]. However, while air pollution exposure is known to occur across an individual's lifetime, at this time, little is known about the consequences of chronic DE exposure in the CNS.

In the current study, we begin to define the deleterious CNS effects in response to subchronic (6 month) DE exposure. More specifically, we address the minimum levels of DE necessary for neuroinflammation, and explore when these exposures are associated with early markers of pre-clinical CNS disease.

## Methods

### Reagents

The α synuclein and GAPDH antibodies were purchased from Millipore (Billerica, MA). The HRP goat anti-rabbit secondary antibody was purchased from Vector Laboratories (Burlingame, CA). TNFα, IL-1β, IL-6, and MIP-1α ELISA kits were purchased from R&D Systems Inc. (Minneapolis, MN). The Tau [pS199] ELISA was purchased from Invitrogen (Carlsbad, CA). All other reagents were procured from Sigma Aldrich Chemical Co. (St. Louis, MO).

### Animals

Ten - twelve week old male Fischer 344 rats (Charles River Laboratories, Raleigh, NC) were housed in 2-m^3 ^whole body chambers (H2000, Hazleton Systems, Maywood, NJ) for a two week acclimation period followed by exposure to filtered air or diesel exhaust (991.8, 311.2, 100.3, 34.9, and 0 μg PM/m^3 ^) for 6 hours a day, 7 days a week, for 6 months. Animals were given water ad libitum throughout the study and fed Teklad certified rodent diet (Harlan Teklad, Madison WI) a *d libitum*, with the exception of when food was removed during the 6 hour exposure period. Rats were euthanized at the end of the 6 month exposures by pentobarbital and each rat received a complete necropsy, including lung lavage. The effect of the DE exposure on health effects independent from the brain are reported elsewhere [30,31]. More specifically, the effects of subchronic exposure on clinical observations, body and organ weights, serum chemistry, hematology, histopathology, bronchoalveolar lavage, and serum clotting factors were shown to be modest [30,31]. Brain tissue was snap frozen and stored at -80C°. For the current study, only one hemisphere of the brain was available for analysis. Housing and experimental use of the animals were performed in strict accordance with the National Institutes of Health guidelines.

### Diesel Exhaust Inhalation Exposure

Diesel exhaust was produced by two 200 model 5.9-L, 6 cylinder Cummins ISB turbocharged diesel engines using certification diesel fuel (371 ppm sulfur, 29% aromatics) and Shell Rotella T, 15 W/40 lubrication oil, as previously reported [22]. The engines were operated on the U.S. Environmental Protection Agency (EPS) heavy duty certification cycle. While recent advances in engine fuel and after-treatment technologies have lowered diesel engine emissions, many older engines that are similar to the model employed for the current study remain in use and are implicated in deleterious health effects associated with heavy traffic [32]. The exhaust was diluted in HEPA and charcoal filtered air to nominally 30, 300, and 1000 μg PM/m^3 ^of total particulate matter (PM), measured by weighing the material collected on glass fiber filters. Actual diesel PM values were later determined to be 992 (High), 311 (Mid High), 100 (Mid Low), 35 (Low), and 0 μg PM/m^3 ^. DE levels reported in the current study span from DE exposure that might be encountered in ambient air near roadways to high occupational levels [22].

Exposure atmospheres were monitored daily for the concentration of PM by sampling of the Pallflex filters (Pall-Gelman, Ann Arbor, MI). Samples were collected hourly for the two highest exposure levels and every 3 hours for the lowest two DE exposures. A single filter sample was collected each day from the control chamber. While the levels of DE in this study are referred to by the net PM mass of each exposure level, the DE is also comprised of multiple additional components, including gases and vapors. This distinction is important, as the nonparticulate components of DE are also noted to have physiological effects [12,33]. The specific composition of the DE exposure has been described in detail previously [22].

### Brain Homogenate Sample Preparation

Olfactory bulb, frontal lobe, temporal lobe, midbrain, and cerebellum were dissected from one brain hemisphere on a cold aluminum block. Each brain region was homogenized in Cytobuster (EMD Chemicals, Gibbstown, NJ) lysis buffer containing Halt Protease Inhibitor Cocktail and Halt Phosphatase Inhibitor Cocktail (Thermo Scientific, Rockford, IL). Samples were spun at 4°C 14,000 g for 5 minutes and supernatant was collected for analysis. Protein concentration was determined by the BCA protein assay (Thermo Scientific, Rockford, IL), per manufacturer instructions.

### Western Blot

Ten micrograms of protein from each midbrain sample was electrophoresed on a 12% SDS-PAGE gel. Samples were transferred to nitrocellulose membranes by semi-dry transfer, blocked with 5% nonfat milk for 1 hr at 24°C, followed by incubation overnight with the anti-GAPDH (1:1000) or anti-α synuclein (1:1000) antibodies at 4°C. Blots were then incubated with horseradish peroxidase-linked mouse anti-rabbit (1:5000) or goat anti-mouse (1:5000) for 1 hr (24°C) and ECL+Plus reagents (Amersham Biosciences Inc., Piscataway, NJ) were used as a detection system. Band density was quantitated with ImageJ [34] and analyzed as a ratio of GAPDH and α synuclein. Results are reported as a percent increase from control.

### TNFα, IL-6, MIP-1α, IL-1β, Aβ42, and Tau [pS199] ELISA

Brain homogenate protein (100 μg/well) from 5 brain regions: the olfactory bulb, the frontal lobe, the temporal lobe, the midbrain, and the cerebellum were assessed for levels of pro-inflammatory cytokines/chemokines and markers of neurodegenerative disease. More specifically, brain region-specific TNFα, IL-6, MIP-1α, and IL-1β levels were measured by ELISA (R&D Systems, Minneapolis, MN), per manufacturer instructions, as previously reported [18]. Temporal and frontal lobe samples were also assessed for the presence of Tau [pS199] by ELISA (Invitrogen, Carlsbad, CA), per manufacturer instructions. The amount of Aβ42 was measured in frontal lobe samples by ELISA with the Human/Rat β Amyloid (42) ELISA Kit (Wako, Richmond, VA), per manufacturer instructions.

### Statistical Analysis

Data are expressed as raw values or the percentage of control, where control values are 100%. The treatment group data are expressed as the mean ± SEM and statistical significance was assessed with a one-way Analysis of Variance followed by Bonferroni's post hoc analysis with SPSS. A value of p < 0.05 was considered statistically significant.

## Results

### Subchronic DE Exposure Elevates TNFα in the Brain: Midbrain Sensitivity

**Figure 1:**
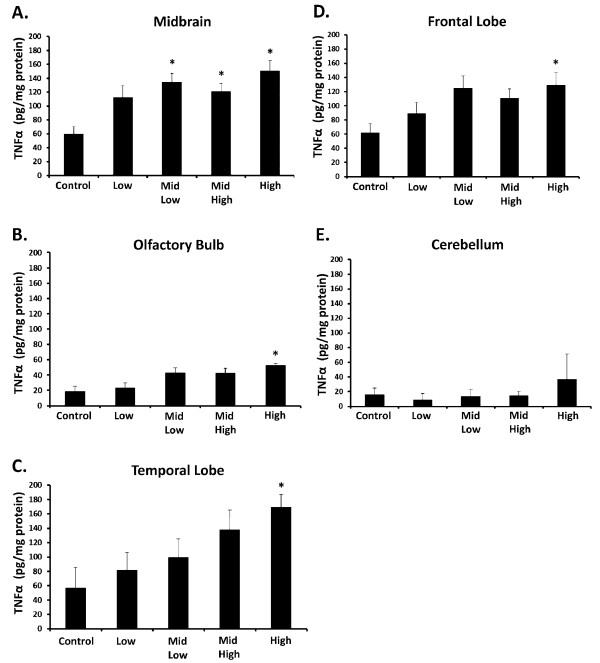
**Subchronic DE Exposure Elevates TNFα in the Brain: Midbrain Vulnerability**. Male Fischer 344 rats were exposed to either filtered air (control, 0 μg PM/m^3 ^DE, n = 8), 35 μg PM/m^3 ^DE (Low, n = 8), 100 μg PM/m^3 ^DE (Mid Low, n = 8), 311 μg PM/m^3 ^DE (Mid High, n = 8), or 992 μg PM/m^3 ^DE (High, n = 8) for 6 months. TNFα protein levels from the (A) Midbrain, (B) Olfactory Bulbs, (C) Temporal Lobe, (D) Frontal Lobe, and (E) Cerebellum were measured by ELISA. An * indicates significant difference (p < 0.05) from control animals. While all components of the brain, with the exception of the cerebellum, showed an elevated TNFα response to DE at some concentration of DE, the midbrain was the most sensitive, producing a significant increase from control at only 100 μg PM/m^3^. = DE.

TNFα is elevated in PD and AD patient brains and has been implicated as a key mechanism of inflammation-mediated neurodegeneration, where the substantia nigra in the midbrain may be particularly vulnerable to its effect [35,36]. We have previously shown that month-long DE exposure significantly elevates TNFα levels in the brain with the largest increase in the midbrain region, but only at the concentration of 2000 μg PM/m^3 ^DE [18]. Here, we measured the effects of lower DE levels and 6 month exposure on 5 brain regions: the olfactory bulb (a hypothesized point of entry of PM in the brain); the frontal lobe (damaged in AD and Frontaltemporal lobe dementia); the temporal lobe (damaged in AD and Frontaltemporal lobe dementia); the midbrain (damaged in PD); the cerebellum (not associated with PD & AD). Results show that all regions with the exception of the cerebellum express elevated TNFα protein levels in response to the highest concentration of DE, 992 μg PM/m^3 ^DE (Figure 1A-E, p < 0.05). However, the midbrain exhibited elevated TNFα levels at 992 μg PM/m^3 ^DE, 311 μg PM/m^3 ^DE, and 100 μg PM/m^3 ^DE (Figure 1E, p < 0.05), indicating a greater sensitivity to the pro-inflammatory effects of DE.

### Subchronic DE Exposure Modifies the Pro-inflammatory Profile of the Midbrain

**Figure 2:**
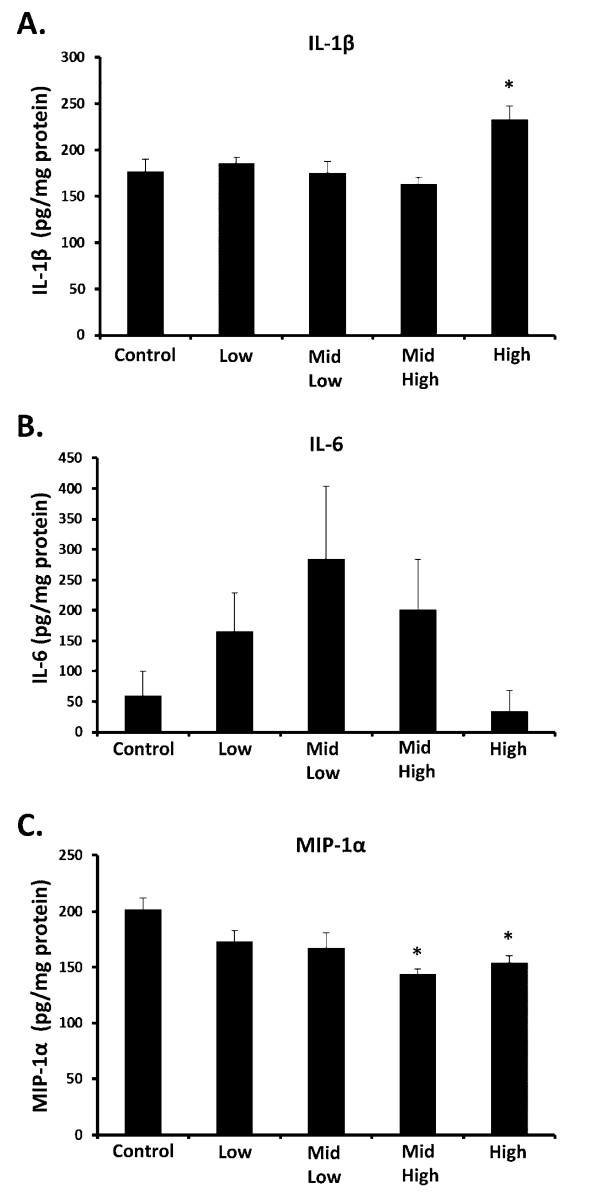
**Subchronic DE Exposure Differentially Regulates Other Cytokines and Chemokines in the Midbrain**. Male Fischer 344 rats were exposed to either filtered air (control, 0 μg PM/m^3 ^DE, n = 8), 35 μg PM/m^3 ^DE (Low, n = 8), 100 μg PM/m^3 ^DE (Mid Low, n = 8), 311 μg PM/m^3 ^DE (Mid High, n = 8), or 992 μg PM/m^3 ^DE (High, n = 8) for 6 months. (A) IL-1β, (B) IL-6, and (C) MIP-1α protein levels were measured in the midbrain by ELISA. An * indicates significant difference (p < 0.05) from control animals. DE elevated IL-1β at only the highest concentration of DE, failed to affect IL-6 levels, and decreased MIP-1α expression in the midbrain.

In an effort to further address the degree of sensitivity of the midbrain to air pollution, we measured the effects of DE inhalation on multiple other pro-inflammatory factors, including cytokines and chemokines. Data reveal that the sensitivity to DE demonstrated with TNFα was not conserved in the response of the pro-inflammatory factors tested. More specifically, IL-6 was not significantly affected (Figure 2B, p > 0.05), IL-1β was only elevated at the highest concentration of 992 μg PM/m^3 ^DE (Figure 2A, p < 0.05), and MIP-1α levels decreased at 311 μg PM/m^3 ^and 992 μg PM/m^3 ^DE (Figure 2C, p < 0.05). Notably, this decrease in MIP-1α levels is consistent with reports on lung effects in the rats, where MIP-1α decreased in lung lavage fluids [31]. Together, these data suggest that longer exposures to air pollution may trigger a compensatory response to neuroinflammation in the midbrain.

### Tau Hyperphosphorylation - DE Elevates Tau [pS199] in the Frontal & Temporal Lobe

**Figure 3:**
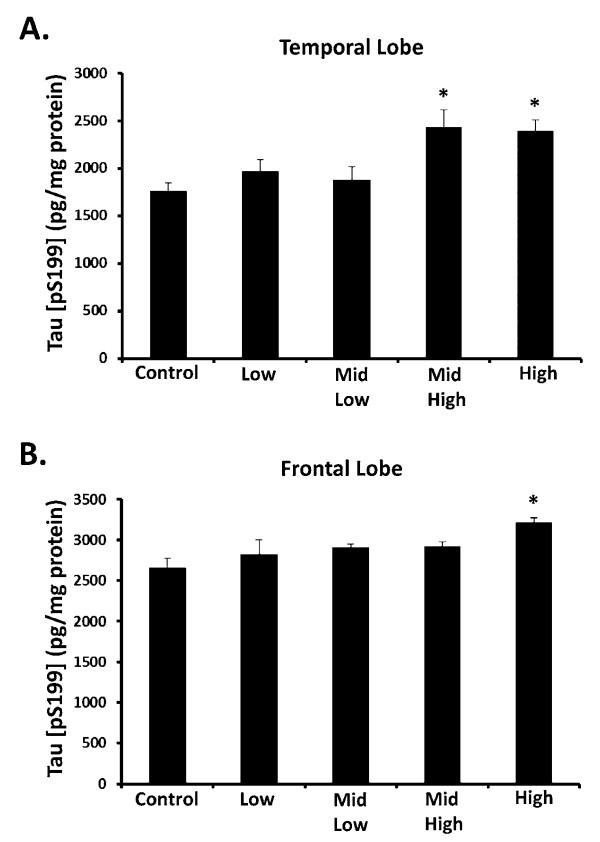
**Subchronic DE Exposure Elevates Tau [pS199] in the Temporal and Frontal Lobes**. Male Fischer 344 rats were exposed to either filtered air (control, 0 μg PM/m^3 ^DE, n = 8), 35 μg PM/m^3 ^DE (Low, n = 8), 100 μg PM/m^3 ^DE (Mid Low, n = 8), 311 μg PM/m^3 ^DE (Mid High, n = 8), or 992 μg PM/m^3 ^DE (High, n = 8) for 6 months. Tau [pS199] protein levels were measured in the (A) Frontal and (B) Temporal lobe by ELISA. An * indicates significant difference (p < 0.05) from control animals. DE elevated Tau [pS199] at the highest concentrations of DE, demonstrating that subchronic exposure to high levels of air pollution is associated with Alzheimer disease-like pathology.

Tau is a microtubule binding protein that promotes microtubule assembly and stability, and as such is expressed in high levels throughout the brain. Tau is linked to AD pathology because it is a major component of the paired helical filaments in neurofibrillary tangles found in AD patient brains [37]. Tau is hyperphosphorylated at several sites during some neurodegenerative diseases, and elevation of Tau phosphorylation at the Ser 199 residue (Tau [pS199]) has been specifically linked to neurofibrillary tangles associated with AD [37]. Importantly, hyperphosphorylation of Tau S199 has also been implicated as an early marker of Tau pathology [38]. Recent reports in humans show that exposure to elevated levels of air pollution is associated with frontal lobe pathology, suggesting that this region is vulnerable [13]. To discern whether DE impacts the phosphorylation of Tau at serine 199, we assessed the levels of Tau [pS199] in both the frontal and temporal lobe, which are affected by AD. Data reveal that Tau [pS199] levels are significantly increased from control at 311 and 992 μg PM/m^3 ^DE in the temporal lobe (Figure 3A, p < 0.05) and only at 992 μg PM/m^3 ^DE in the frontal lobe (Figure 3B, p < 0.05). Consistent with human findings investigating urban air pollution [13], our data confirm that subchronic DE exposure elevates subclinical markers and induces AD-like pathology in both the frontal and temporal lobe.

### DE Elevates α Synuclein

**Figure 4:**
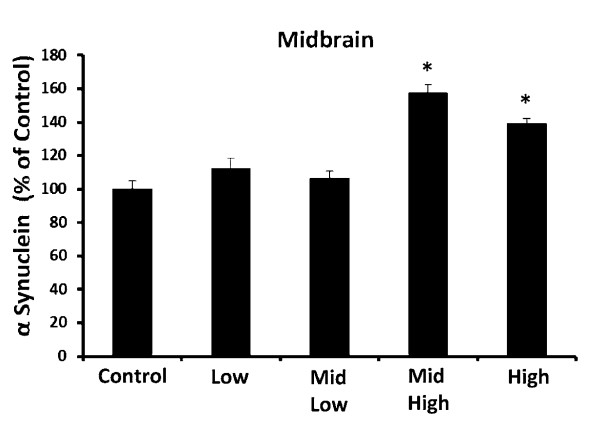
**Subchronic DE Exposure Elevates α Synuclein in the Midbrain**. Male Fischer 344 rats were exposed to either filtered air (control, 0 μg PM/m^3 ^DE, n = 8), 35 μg PM/m^3 ^DE (Low, n = 8), 100 μg PM/m^3 ^DE (Mid Low, n = 8), 311 μg PM/m^3 ^DE (Mid High, n = 8), or 992 μg PM/m^3 ^DE (High, n = 8) for 6 months. α Synuclein protein levels were measured in the midbrain by western blot. An * indicates significant difference (p < 0.05) from control animals. DE elevated α synuclein protein levels in the midbrain at only the highest concentrations tested, demonstrating that subchronic exposure to high levels of air pollution is associated with Parkinson's disease-like pathology.

Recent evidence points to α synuclein as more than merely a hallmark protein found in Lewy bodies in PD. For example, excessive elevation of wild type α synuclein (SNCA) due to genetic multiplication causes early onset, autosomal dominant-familial PD [39]. In addition, recent studies have also demonstrated that α synuclein is elevated in the midbrain of sporadic PD patients [40]. In fact, α synuclein elevation is believed to occur early in PD progression and its use has been proposed as a pre-clinical marker of PD [41]. Interestingly, previous studies in humans from highly polluted areas show an elevation of brain α synuclein [13,42]. Consistent with reports on post mortem analysis of PD patient brains and those exposed to high levels of air pollution, we show in the current study that 992 μg PM/m^3 ^DE results in significant elevation of α synuclein protein in the midbrain (Figure 4, p < 0.05), as measured by western blot analysis. Thus, here we demonstrate that high concentrations of air pollution elevate markers of PD pathology in rats.

### DE Elevates Aβ42

**Figure 5:**
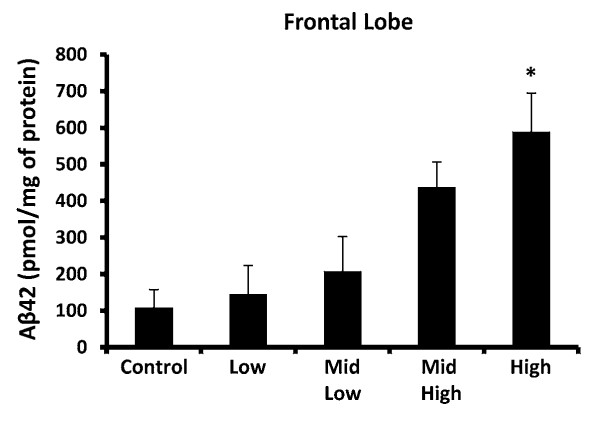
**Subchronic DE Exposure Elevates Aβ in the Frontal Lobe**. Male Fischer 344 rats were exposed to either filtered air (control, 0 μg PM/m^3 ^DE, n = 8), 35 μg PM/m^3 ^DE (Low, n = 8), 100 μg PM/m^3 ^DE (Mid Low, n = 8), 311 μg PM/m^3 ^DE (Mid High, n = 8), or 992 μg PM/m^3 ^DE (High, n = 8) for 6 months. Aβ42 protein levels were measured in the frontal lobe ELISA. An * indicates significant difference (p < 0.05) from control animals. DE elevated Aβ42 protein levels in the frontal lobe at only the highest concentrations tested, demonstrating that subchronic exposure to high levels of air pollution is associated with Alzheimer's disease-like pathology.

Aβ42 occurs due to aberrant processing of the amyloid precursor protein [43]. Unlike other isoforms, Aβ42 easily aggregates, is a major component of plaques, and has been widely implicated in AD and frontotemporal dementia (FTD) pathology [43]. In fact, deposition of Aβ42 is linked to cognitive changes and may even be a marker for AD [43,44]. Importantly, previous studies have shown that people living in highly polluted cities have elevated brain levels of Aβ42, when compared to people living in less polluted regions [14], suggesting that air pollution may be causing AD-like pathology. Here, we show that that subchronic exposure to 992 μg PM/m^3 ^DE in rats results in a significant increase in the amount of Aβ42 accumulation in the frontal lobe (Figure 5, p < 0.05), indicating an elevation of an AD-like and FTD - like marker.

## Discussion

Accumulating evidence indicates that the brain detects and responds to diverse classifications of inhaled air pollution, such as metals, ozone, urban PM, and DE with a common pathway of neuroinflammation [10]. However, it is unclear whether the pro-inflammatory response in the brain is merely a marker of exposure to air pollution or whether this response is linked to more sinister consequences. Here, we begin to explore these questions using subchronic DE exposure in an effort to model the persistent nature of air pollution exposure and employ the use of lower levels that are comparable to busy road-way and occupational levels. Together, this approach allowed us to begin to address what conditions are necessary for air pollution to elicit CNS effects and assess whether markers of neurodegenerative disease pathology occur with neuroinflammation.

TNFα is a "potent" pro-inflammatory cytokine elevated in both AD and PD patients, where it is implicated to play a causal role in neurotoxicity [45]. Consistent with previous reports on short term and high exposures to air pollution [18,28,46] and chronic human studies [14], here we show a general pro-inflammatory response in the brain with subchronic DE exposure, which we propose may be due in large part to a systemic/peripheral effect that reaches the entire brain, rather than solely through the olfactory bulb, a favored pathway of PM entry into the brain [47,48]. This is evidenced by the fact that the olfactory bulb showed a blunted TNFα response when compared to other regions and TNFα levels were elevated in most regions tested, with the exception of the cerebellum (Figure 1). The cerebellum contains fewer numbers of the brain's resident innate immune cell, microglia [49], and it is not traditionally involved in AD or PD pathology. Thus, consistent with prior reports [18], our current data also support that microglia may regulate the brain region-specific pro-inflammatory response to DE.

More specifically, our previous work with short term (1 month) inhalation of higher levels of DE indicated that the midbrain, which contains the substantia nigra damaged in PD, is more vulnerable to the pro-inflammatory effects of DE [18]. In particular, the midbrain produced the most robust elevation of multiple cytokines, chemokines, and nitrated protein levels when compared to other brain regions [18]. Consistent with this premise, analysis of microglial markers confirmed that the midbrain expressed highest levels of microglial markers at rest in control animals and showed the greatest elevation or microglial markers in response to short term and high DE exposure [18]. Interestingly, in response to subchronic DE in the current study, the midbrain expressed TNFα levels comparable to the other brain regions tested (Figure 1A), suggesting that perhaps the pro-inflammatory response may be tempered with longer exposures. However, the midbrain was the only region to show significantly elevated TNFα levels in response to lower levels of DE (100 μg PM/m^3 ^) with 6 month exposure (Figure 1A), demonstrating that the midbrain sensitivity to air pollution extends to longer and lower DE exposures.

We next sought to discern whether this enhanced sensitivity to DE in the midbrain generalized to other pro-inflammatory markers. IL-1β is another pro-inflammatory factor elevated in PD and AD that has been widely implicated in neuronal damage [50]. Here, we show that IL-1β levels are elevated in response to subchronic DE, but only at the highest concentration of 992 PM μg/m^3 ^(Figure 2A, p < 0.05). IL-6 is both a beneficial and potentially detrimental cytokine that responds to neuronal damage and is elevated in AD and PD [51]. However, we found no significant effect of IL-6 in the midbrain in response to DE (Figure 2B, p > 0.05). MIP-1α is a chemokine important for microglial migration [52] and our current study demonstrates that subchronic DE exposure causes a reduction in MIP-1α in the midbrain at the highest concentrations tested. This decline in MIP-1α is consistent with a pattern seen in the lung of these same animals, as previously reported [30]. Thus, the enhanced sensitivity seen with TNFα in the midbrain at lower concentrations of DE is not conserved across all pro-inflammatory factors tested, which is different than what we had previously reported with one month DE exposure [18]. This suggests that perhaps compensatory mechanisms are triggered with longer exposures. Together, the data support that TNFα may be an important cytokine for the CNS effects of air pollution.

Several human studies have shown that chronic exposure to high levels of air pollution is linked to AD-like pathology, including elevation of diffuse plaques, neuroinflammation, and frontal lobe damage [13,14,42]. Given that neuroinflammation, particularly elevation of TNFα, has been linked to the induction of hyperphosphorylation of Tau [53], we sought to determine whether DE had an effect on this parameter in a subchronic inhalation rat model. Tau is a major component of neurofibrillary tangles found in AD and FTD patient brains where it is hyperphosphorylated at several sites, including the Ser 199 residue (Tau [pS199]) [37]. Further, hyperphosphorylation of Tau S199 has been implicated as an early marker of Tau pathology [38] We show here, that only the highest level of DE caused elevation of Tau [pS199] in the frontal lobe (Figure A, p < 0.05) and temporal lobe (Figure 3B, p < 0.05). In addition, we also show that only the highest level of DE caused elevation of Aβ42 (Figure 5, p < 0.05). These findings support that high levels of DE may be linked to neuropathology associated with pre-clinical AD and FTD markers.

Previous studies in humans from highly polluted areas show an elevation of brain α synuclein [13,42]. However, our earlier reports employing only month-long DE exposure show robust neuroinflammation with no significant effect on α synuclein levels or evidence of neurotoxicity in the midbrain [18]. Here, we explored whether DE exposure elevated α synuclein in response to longer, subchronic DE exposure. α Synuclein is known to be elevated in the midbrain of sporadic PD patients [40], where elevation occurs early in the disease and its use has been implicated as a pre-clinical marker of PD [41]. In the current study, we show that DE increased α synuclein levels at only highest concentrations (Figure 4. p < 0.05).

## Conclusion

Together, these results show that 6 month exposure to DE elevated TNFα in most brain regions tested, with the exception of the cerebellum. In particular, the midbrain region, which houses the substantia nigra that is selectively lost in PD, was the most sensitive to DE effects, as TNFα was elevated in response to low levels of DE (100 μg PM/m^3^). There was also evidence of compensatory mechanisms in the midbrain with subchronic DE exposure, as IL-6 was not significantly altered, IL-1β was only elevated at the highest concentration, and MIP-1α decreased at higher concentrations in the midbrain. Tau [pS199], a protein modification linked to both AD and FTD, was elevated at only the highest concentrations of DE in both the temporal and frontal lobes. Aβ42, a protein implicated in both AD and FTD pathology, was also increased in the frontal lobe in response to DE only at the highest concentration. Interestingly, α synuclein was elevated in the midbrain at only the highest concentration, suggesting that the TNFα increase at lower concentrations is not yet sufficient to initiate this potential marker of preclinical PD. These findings indicate that while some compensatory mechanisms may occur, the neuroinflammatory response to air pollution, particularly the TNFα response, is still present with subchronic exposure and may precede evidence of neuropathology. Future research needs to address the effects of lifetime air pollution exposure and the impact of aging on neuroinflammation and neurotoxicity.

## List of abbreviations

DE: diesel exhaust; PM: particulate matter; PD: Parkinson's disease; AD: Alzheimer's disease; DA: dopamine; TH: tyrosine hydroxylase; TNFα: tumor necrosis factor alpha; IL-1β: Interleukin 1 beta; IL-6: Interleukin 6; MIP-1α: Macrophage inflammatory protein 1 alpha; NAAQS: National Ambient Air Quality Standards; Aβ: Beta Amyloid; FTD: Frontotemporal dementia.

## Competing interests

The authors declare that they have no competing interests.

## Authors' contributions

SL homogenized the brain samples, calculated protein concentrations, ran ELISAs, and completed most of the experiments for these studies. MJS ran the gels and did the densitometry for the midbrain α synuclein concentration. JM ran the animal experiments and collected brain tissue. MLB performed statistical analyses and wrote the manuscript. All authors contributed conceptually to the writing of the manuscript and approved the manuscript.
